# Selection criteria for foaming agent and mechanical performance evaluation of conditioned soil for EPB shield tunneling in water-rich sand strata

**DOI:** 10.1038/s41598-026-38868-y

**Published:** 2026-03-05

**Authors:** Yuhang Zhou, Bitang Zhu, Ruping Luo, Jin Yu, Qijing Yang, Sanjay Nimbalkar

**Affiliations:** 1 School of Civil Engineering and Architecture, East China Jiaotong University, Nanchang, 330013 China; 2 Engineering Research & Development Centre for Underground Technology of Jiangxi Province, Nanchang, 330013 China; 3 School of Civil and Environmental Engineering, University of Technology Sydney, Sydney, NSW 2007 Australia

**Keywords:** EPB shield tunnelling, Soil conditioning, Foaming agent, Foam-conditioned sand, Water-rich sand strata, Engineering, Civil engineering

## Abstract

The selection of a suitable foaming agent for soil conditioning in the Earth Pressure Balance (EPB) shield tunnelling through water-rich sand strata currently lacks a standardized test method and mechanical indices. This paper presents systematic investigations on several key factors influencing the soil conditioning of EPB shield tunnelling regarding foam. The mechanical properties of the foaming agent solution and foam have been assessed for eight different foaming agents through a series of specifically devised laboratory tests. The efficacy of the foaming agent solution has been validated through laboratory tests on conditioned water-rich sandy soil for three types of foaming agent solutions. Furthermore, a standardized method has been proposed for selecting foaming agent and a mechanical characterisation scheme for conditioned soil of EPB tunnelling in water-rich sand strata. Experimental results indicate that the optimum foaming concentration should be controlled to be at 3% for EPB shield tunnelling in water-rich sand strata. At an optimal foaming concentration of 3%, the investigation test results indicate the following mechanical indices of foaming agent should be satisfied: 1) surface tension < 40 mN/m at the critical micelle concentration; 2) the foam volume > 150 mL after 15 min with the Roche foam meter; 3) the foam expansion ratio under the atmospheric pressure > 12; and 4) the half-life time > 400 s. Furthermore, the concave decay type of foam (class II) is preferred over the linear decay type (Class I), as confirmed by the tests on the mechanical performance of foam conditioned sand. It has also found that the conditioned soils having indices with the slump of 150–200 mm, permeability coefficient k < 10–5 m/s, and undrained shear strength of 3–7 kPa, work well for EPB shield TBM tunnelling through sandy soils in Nanchang.

## Introduction

EPB shield and slurry shield have emerged as the two most used tunnel boring machines for constructing urban metro tunnels^1–5^. However, due to the drawbacks of significant environmental pollution, high costs and construction challenges, the slurry shield is typically only utilized in certain river-crossing or undercrossing tunnels^6–8^. EPB shield tunnelling is the most common construction method in urban metro tunnels, but it is usually necessary to inject additives (e.g. foam, bentonite slurry, polymer) to the excavated muck in front of the cutterhead or inside the excavation chamber to achieve appropriate plasticity and fluidity, to ensure the stability of the tunnel face and smooth discharge of the excavated muck^9–13^. During tunnel boring through the widely distributed water-rich sand strata with a high-water head, the excavated muck is easy to cause gushing at the screw conveyor because of its high permeability. The excavated muck, having low viscosity and high internal friction angle, tends to develop soil arching, which hinders smooth discharge of spoil and increases energy consumption^14,15^. Therefore, the use of foam, bentonite slurry, and polymer is often required to enhance the performance of the excavated muck. The purpose is to reduce the tunnel boring machine (TBM) thrust and cutterhead torque, lower shear strength and water permeability of the excavated muck, thus improve its flow characteristics and workability to facilitate smooth tunnelling^16,17^.

Foam is one of the most widely used soil conditioners as it is cheap and handy. The foam is generated by a foaming device when a foaming agent is mixed with water and air. The quality of foam greatly impacts the effectiveness of soil conditioning. To better understand the mechanism of foaming agents, many researchers have studied various factors affecting foam properties. For instance^18^ developed laboratory foam preparation devices and studied the factors including foam firing gun type, foam volume flow, conveying pipeline length, foaming agent type, foaming agent concentration on the foam density, liquid discharge time and bubble size, and the foamability and stability of the foam were systematically evaluated^19^ used laboratory experiments to determine the main properties of coarse particle excavation muck and carried out soil conditioning tests on it. A large number of fundamental experiments have proved that foam and other conditioners are necessary for soil conditioning^20^ studied different ionic types of surfactants, the main component of foam, and divided them into anionic, cationic, amphoteric and non-ionic surfactants according to different hydrophilic base charges from a micro mechanism persective^21^ pointed out that anionic surfactants, which have a negatively charged hydrophilic base, are suitable for most formations and are currently the most widely used for soil conditioning. Generally, the surface of clay particles has a positive charge. Anionic surfactants have a hydrophilic base that is adsorbed onto the surface of soil particles, while the hydrophobic base remains distant from the surface of soil particles. These characteristics lead to an increase in free water content and fluidity, enhancing the flow plasticity of the clayey excavation muck. Additionally, anionic surfactants generally exhibit higher foamability than others. Nonionic surfactants, with a chargeless hydrophilic group, are commonly utilized in soils with varying PH levels, whereas cationic and amphoteric surfactants find less application^22^ formulated a new foaming agent and verified its effectiveness in soil conditioning for EBP shield tunnelling by comprehensive laboratory tests, including a mixing test, friction coefficient test, adhesion resistance test, slump test and direct shear test.

A series of characterisation tests was conducted by many researchers to evaluate the key physical properties of foam and its performance in soil conditioning for EPB shield tunnelling. However, there is still a lack of a unified understanding of the selection index and testing method of foaming agents suitable for the soil conditioning of shield tunnelling in water-rich sand strata^23–26^. As a result, it is necessary to select an appropriate foaming agent through a large number of trial mixing tests in engineering practice. The selection of a foaming agent determined solely based on cost, can result in poor conditioning effect and cause accidents such as TBM cutting face instability, excessive deformations and even ground collapse. In addition, it is also necessary to standardize the selection and injection rate of soil conditioners so as to automatically adjust soil conditioning during EPB tunnelling with the development of intelligent driving technology^27,28^. Therefore, it is of great significance to develop the standard test method of the foaming agent performance for soil conditioning and quantify the performance parameters of the foaming agent for EPB tunnelling.

In this study, a series of laboratory tests have been conducted to evaluate the key performance parameters of eight commercial foaming agents. Types of foaming agents were classified based on the crucial indices for foam stability. Further mechanical property tests were carried out on the conditioned sandy soils to validate the surface tension, foaming capacity, foaming rate, and half-life time for the classified foaming agent types. This study aims to propose a standard method of determining the physical indices of the foaming agent for soil conditioning, and performance validation of the mechanical characterisation of the conditioned muck in front of the cutterhead or within the excavation chamber of EPB shield tunnelling in the water-rich sand strata.

## Test programs on various foaming agents

### Test programme

In this experiment, eight commercial foaming agents currently used in shield tunnelling projects were selected as the research objects, labeled as A to H respectively. The reason for choosing these foaming agents was that they covered various types such as synthetic, natural, and special composite ones, and there were significant differences in their chemical composition, foaming mechanism and expected performance, enabling a systematic evaluation of their improvement effects on water-rich sandy layers^29,30^. A series of tests on the surface tension, foaming ratio test, foaming capability and stability, and foam half-life were carried out for eight kinds of foaming agents used for shield tunnelling in water-rich sand strata. Through implementing a comprehensive test program in this study, a standard testing method has been proposed for the foaming agent used for soil conditioning of the sandy soil muck generated in the TBM working chamber. Based on the test results standard procedures for optimization of the foaming agent for practical applications have also been proposed.

### Basic principle of foam generation and design of preparation system

**Fig. 1:**
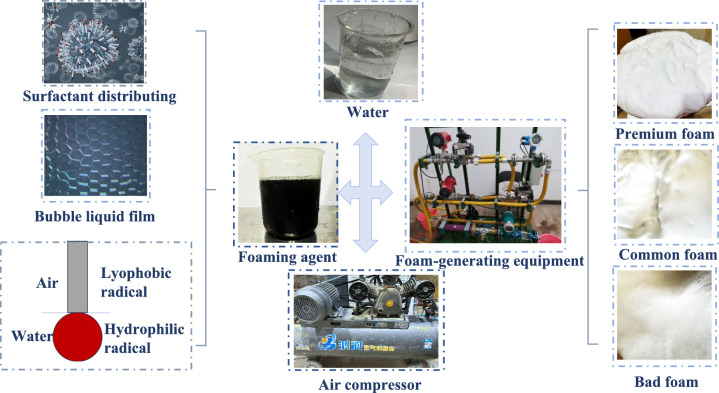
Schematic diagram of foam generating.

The foaming apparatus used in this study, and the process and microscopic mechanism of the foaming agent are shown in Fig. 1. Foam is a typical two-phase (gas–liquid) system, which is produced by the mixed solution of foaming agent and water and compressed air through the foam generator, of which about 90% is compressed air and about 10% is foaming agent solution as revealed by previous researchers^31–34^. The main component of the foaming agent is surfactant, which is composed of long molecular chains formed by polymerisation and contains hydrophilic and hydrophobic groups. When the foaming agent combines with water, the hydrophilic portion of the surfactant dissolves in water, while the hydrophobic part remains insoluble, floating on the solution’s surface, absorbed at the gas–liquid interface, creating a durable film. This film reduces the surface tension between the gas–liquid two-phase system, making it easier to foam as the surface tension decreases. Once the foaming agent solution is mixed with compressed air with a foam generator, bubbles are produced by the liquid film and the gas enclosed within it. The gas–liquid surfactant molecules are oriented in a specific manner (The hydrophobic group is inside the bubble and the hydrophilic group is outside the bubble). These surfactant molecules adsorbed on the surface of the bubbles have a repulsive force that prevents the accumulation and loss of water molecules outside the adjacent bubbles, resulting in a stable, uniform, and fine foam as found by some researchers^35,36^.

**Fig. 2:**
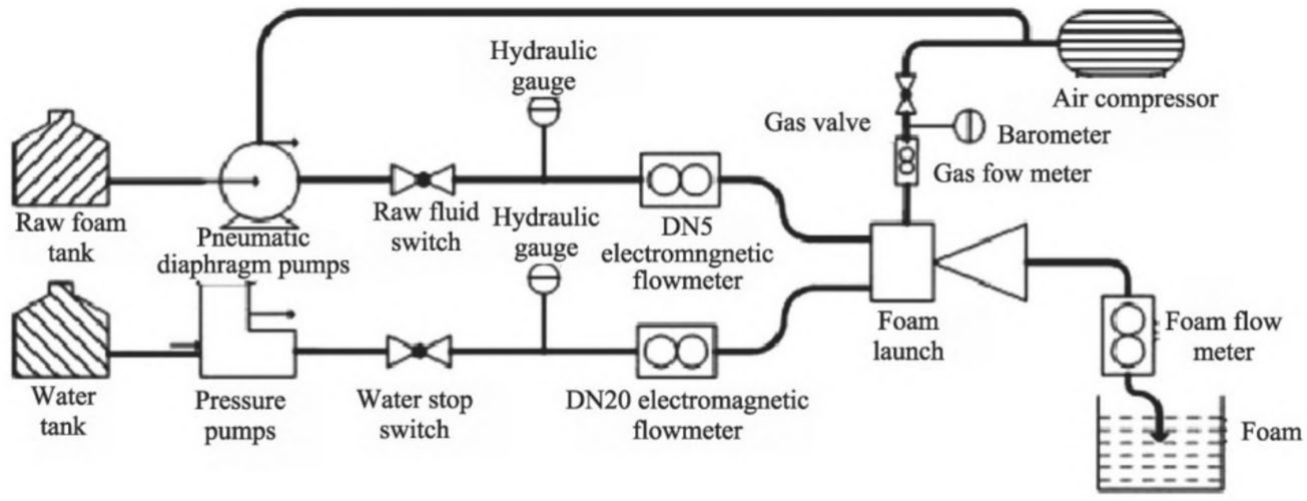
Flow chart of foam generator system.

In this study, a self-designed foam generator was used to prepare the foam required for the test. Under the action of a pneumatic diaphragm pump, the stock liquid of the foaming agent entered the foam generator successively through the stock liquid switch, hydraulic meter, and DN6 electromagnetic flowmeter. Under the action of the hydraulic pump, water entered the foam generator through the water stop switch, hydraulic meter, and DN20 electromagnetic flowmeter in turn. The air compressor provided compressed air, which is used to pump the foaming agent stock solution and in turn through the air stop valve, DN20 gas flow meter, and barometer. The foam stock solution and water were dispersed by the turbulent pump in the foam generator under the action of compressed air, and the foam was exported through the outlet pipe. The maximum gas pressure provided by the air compressor could reach as high as 0.8 MPa, which is consistent with the maximum pressure of the soil conditioning system of the shield machine, and the electromagnetic flowmeter was used to measure the velocity and flow of the liquid. The main working principles of the foam preparation system is shown via the flow chart in Fig. 2.

### Surface tension test

**Fig. 3:**
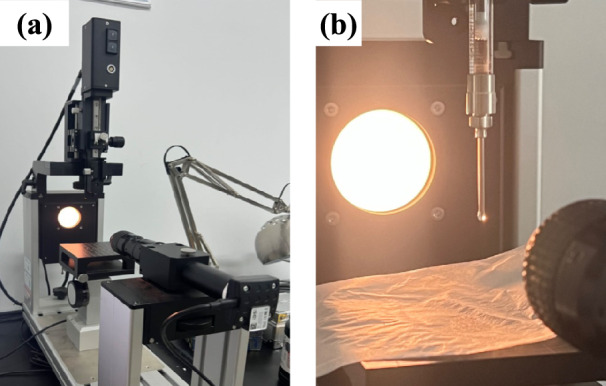
Measurement of surface tension of foam solution by a video OCA measuring instrument: (**a**) video OCA measuring instrument (**b**) Test set-up.

The surface tension of the foaming agent solution is an important factor in evaluating the foaming capability and stability of foaming agent. According to Laplace’s formula, the pressure difference of the flat film at the gas–liquid junction is proportional to the interfacial tension, and the smaller the interfacial tension, the smaller the pressure difference at the junction, and the easier it is to foam^37^. In this study, a video Optical Contact Angle (OCA) measuring instrument-Pendant drop was used to measure the surface tension. The test equipment and test set-up diagram are shown in Fig. 3. In this study, the surface tension was measured by the hanging drop method, which uses a needle tube to inject a large enough droplet into the camera, and then calculates the surface tension by fitting the Young–Laplace equation, i.e.:

1 $$\sigma = \Delta \rho g(ds)^{2} /H,H = H(ds/de)$$

Where $\Delta \rho$ is the density difference; *g* is the gravitational acceleration; *d*e is the length of the droplet with the largest diameter after it reaches its maximum; Take this length as the height of the droplet, and make a diameter at the vertex, whose length is *d*s; *H* is a function of *d*s and *d*e.

**Fig. 4:**
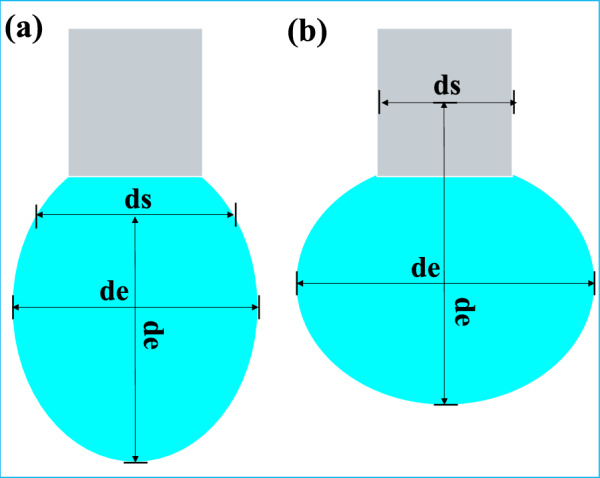
Measurement principle and error diagram: (**a**) When the droplets are large enough (**b**) When the droplets are too small.

It is worth noting that the density difference *Δρ* and gravitational acceleration *g* are unchanged during the test, and the variable is ${ds}^{2}/H$. Therefore, if the droplet injected by the needle is too small during the test, *d*e will be longer than the total length of the droplet, and the value of *d*s will be taken on the injection needle. This makes the test data wrong, so the drop injected must be large enough. The measurement principle and error diagram are shown in Fig. 4.

### Foaming ratio test

**Fig. 5:**
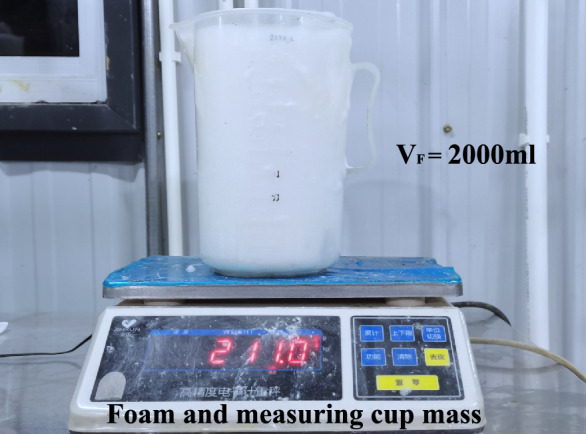
Test set-up to evaluate foam expansion ratio.

When foam is used for soil conditioning of EPB shield tunnelling, the main performance indices of foam are the foaming ratio of foam solution and foam half-life, the former reflects the economy of foam, and the latter reflects the stability of foam. Foam Expansion Ratio (*FER*) refers to the foam produced per unit volume of foaming agent solution. The foaming ratio under atmospheric pressure in the laboratory can be measured directly by a measuring cylinder, and the foaming volume can be measured after the amount of foaming agent solution passing through the foaming generator. Ignoring the mass of the gas in the foam, the mass of the foam is the total mass of the foam agent solution, and the density of the foam agent solution is about 1.0 g/ml. The foaming ratio test apparatus of the foaming agent is shown in Fig. 5, and the *FER* is determined as follows:

2 $$\mathrm{FER} = \frac{{\mathrm{V}}_{\mathrm{F}}}{{\mathrm{V}}_{\mathrm{L}}}$$

where: ${\mathrm{V}}_{\mathrm{F}}$ is the foam volume; ${V}_{L}$ is the volume of foaming agent solution

### Foaming capability and stability test

**Fig. 6:**
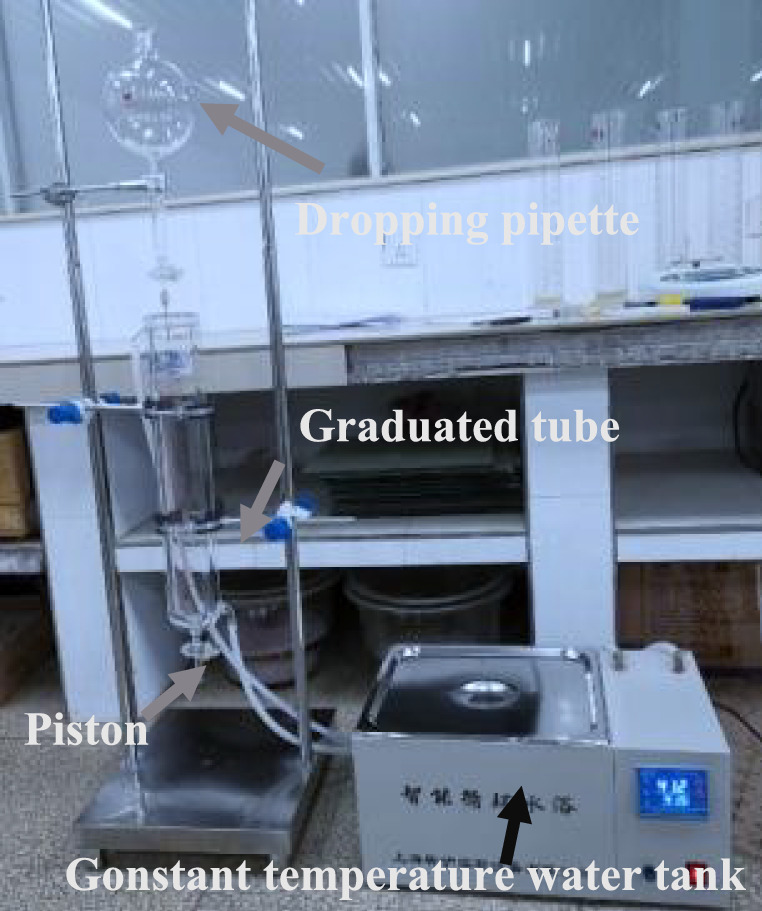
Rothschild foam apparatus.

During the actual construction process, it is crucial to pump the generated foam from the foaming system into the cutter head chamber of the shield machine to ensure full contact with the excavated muck to achieve the desired conditioning effect. Foam loss is often associated with the foam transportation and soil conditioning process, making the foam survival time a critical factor. In this study the Rothschild foam apparatus was utilized to assess foam stability and foaming capability. The foaming capability of a foaming agent solution was determined using the Rothschild foam apparatus, as depicted in Fig. 6. The apparatus used was a glass tube having an inner diameter of 50 mm and a graduated tube length of 700 mm. During experiment 200 ml of foaming agent solution was released from a drop tube at a specific vertical height, the foam generation activities were observed in the centre of the scale tube, and the foam volume and its changes were measured. The foam volumes were recorded at 0.5 min, 3 min, 5 min, 10 min, and 15 min intervals respectively.

### Foam half-life test

The effectiveness of the foam injected into the excavation chamber depends on its stability under agitation. During the EPB tunnelling, the foam experiences a series of processes, including foam formation in the foam preparation system, mixing with muck at the cutter head and inside the excavation chamber, and discharge of muck from the screw conveyor. If the foam is not stable and prone to bursting, the conditioning effect will be compromised. Foam stability is crucial as it determines how long the foam can survive without bursting under static conditions. If the foam is not stable under static conditions, it is likely to be destroyed under agitation, hindering the uniform lubrication of the excavated muck, especially in water-rich sand strata.

**Fig. 7:**
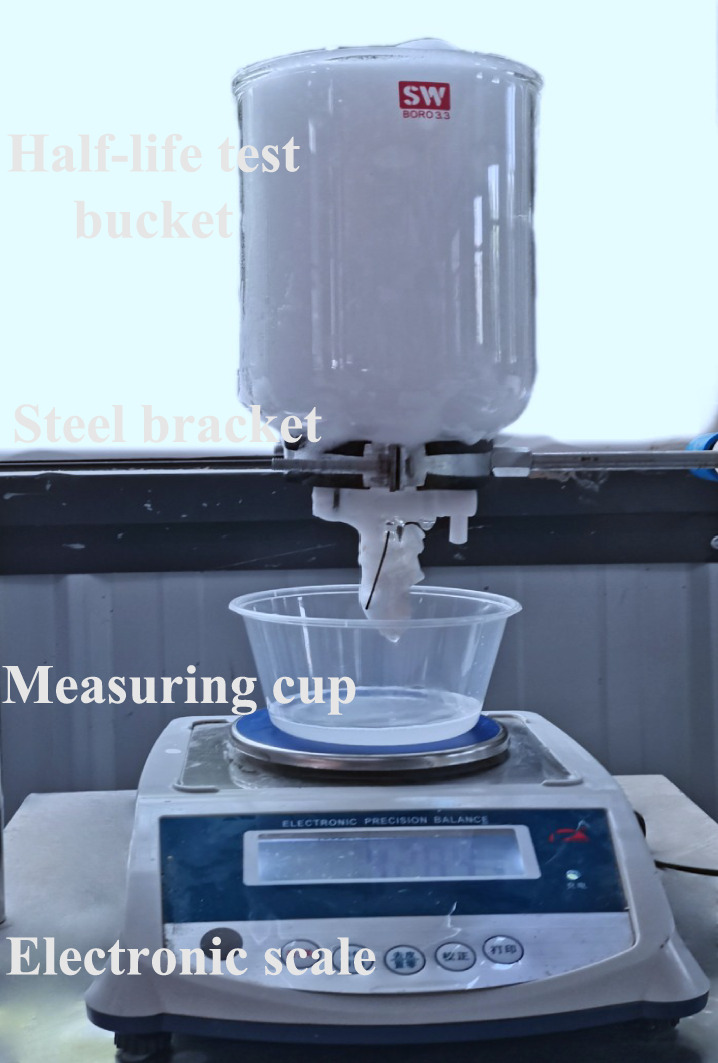
Roche foam meter for foam half-life measurement.

Foam stability is generally characterized by half-life. The foam half-life is measured with the following instruments: fading cylinder; steel bracket; measuring cup; small electronic weighing scale (accuracy is 0.01 g). During the test, the droplet tube and the electronic scale were supported by a steel bracket, and the measuring cup (as shown in Fig. 7) was placed on a small electronic weighing scale (with an accuracy of 0.1 g) to measure the burst into liquid foam. The test steps carried out are described as follows:

1. Foam that meets the experimental requirements were generated with a foaming agent solution of different concentrations. A certain volume of foam was quickly measured into the droplet tube and placed on the steel support.

2. The reading ${m}_{foam}$ taken on the electronic scale at a time was defined as the foam mass. The piston was opened, and the stopwatch was pressed. The mass of liquid extracted from the foam was measured at intervals of 30 s. The foam-fading liquid in the droplet tube flowed into the graduated cylinder. When the reading on the small electronic scale was equal to ${m}_{foam}\mathrm{/2}$, the time t (s) passed from the starting time was recorded, which is the foam half-life.

## Test results and selection criteria of foaming agent

### Surface tension of foaming agent solution

**Fig. 8:**
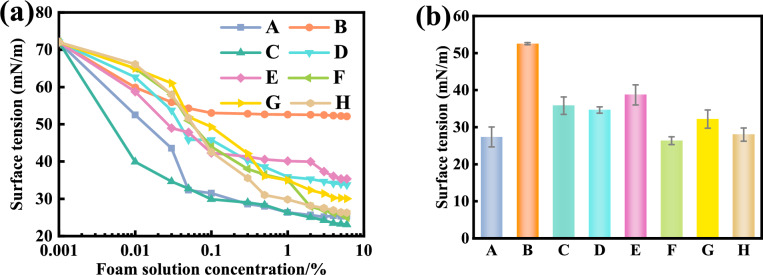
Surface tension of foaming agent solution varying with the concentration: (**a**) Surface tension with concentration; (**b**) Surface tension at the critical micelle concentration.

Before testing the surface tension of 8 commercial foaming agent solution, the OCA measuring instrument was calibrated by measuring the surface tension of pure water at room temperature. This was measured as 72 mN/m, which is very close to the theoretical pure water surface tension of 72.8 mN/m, with an error of only 1%. Fig. 8(a) shows the surface tension values of all foaming agent solutions tested with varying concentrations. It can be seen that:

1. The surface tension of all foaming agent solutions decreases sharply at the beginning. The foaming agent C experiences the quickest drop in the surface tension, from 72 mN/m of pure water to 39 mN/m at the concentration of 0.01%, followed by the foaming agent A. The foaming agents D, E, F, G and H are of similar drop trend, while the foaming agent B has the slowest drop with the largest tension of 53 mN/m at the concentration of 0.1%.
2. There is a critical concentration beyond which the surface tension of the solution will drop gradually as the concentration increases, which is called the critical micelle concentration. The surface tension values at the critical micelle concentration of the 8 kinds of foaming agents tested are shown in Fig. 8(b). The foaming agent E has the largest critical micelle concentration of 2%, and the foaming agent A has the smallest value of 0.05%. The surface tension is 53 mN/m for the foaming agent B, followed by the foaming agent E (39 mN/m), D (36 mN/m), A (32 mN/m), G (31 mN/m), H (30.5 mN /m), C (29.5 mN /m) and F (28 mN/m), respectively.
3. All surface tension-concentration curves tend to be stabilised when the concentration is beyond 2%-3%. Thus, a concentration of 3% is recommended for the selected foaming agent.

Foam-forming molecules have an amphiphilic structure, meaning one part of the molecule is hydrophilic and the other part is hydrophobic. When added to water, they spontaneously adsorb onto the water–air interface (i.e., the water surface). This adsorption alters the arrangement of water molecules at the interface, reducing the interfacial tension. As the foam-forming agent (surfactant) is initially added to water, surfactant molecules gradually accumulate at the water surface, forming a monolayer adsorption film that reduces the interaction between water molecules, thereby lowering the surface tension. As the concentration of the foam-forming agent increases, more surfactant molecules are adsorbed at the interface, further reducing the surface tension. When the concentration of the foam-forming agent reaches a certain threshold, the critical micelle concentration (CMC), the surfactant molecules no longer simply adsorb at the interface but start to aggregate in the solution to form micelles. Micelles are spherical structures formed by the aggregation of surfactant molecules, with their hydrophobic tails pointing towards the interior of the micelle and their hydrophilic heads facing the external aqueous solution. At this point, the water surface is no longer affected by the continued adsorption of foam-forming agent molecules, and thus the surface tension remains at a low and stable level. As foam generation is closely relevant to the low surface tension, the foaming agent C is inferred to have the highest efficiency in the foam generation, followed by the foaming agent F, C, H, G, A, D and E. The foaming agent B is not recommended due to its high stabilised surface tension. Generally, the foaming agent should be of surface tension less than 40 mN/m at the critical micelle concentration and the optimum concentration is 3%. It should be further pointed out that the selection of foaming agent shall be considered comprehensively from the aspects of economy and foam stability.

### Foaming ratio of foaming agent solution

**Fig. 9:**
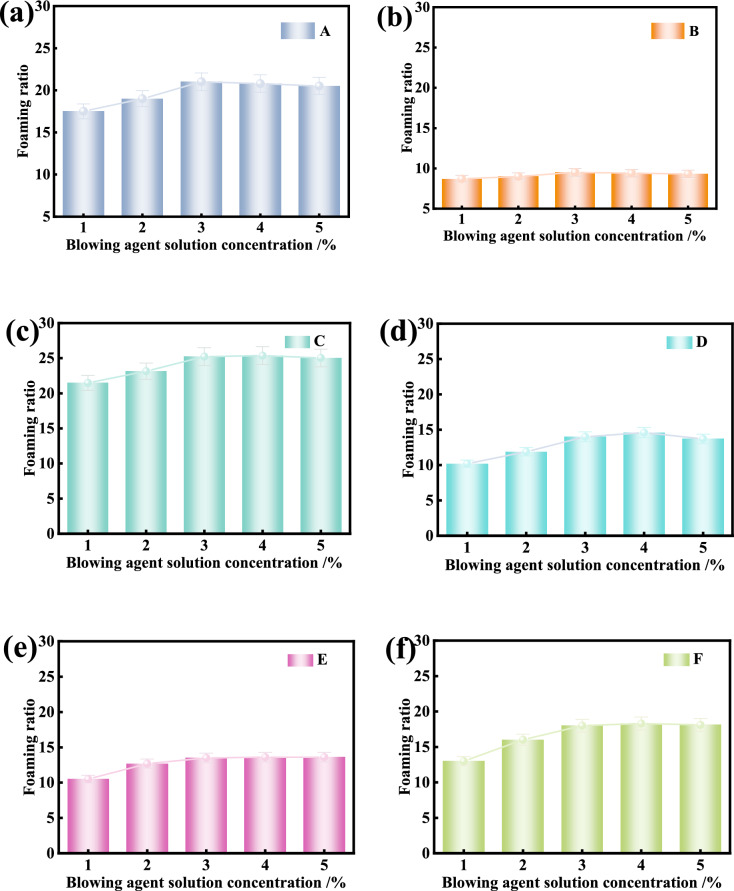

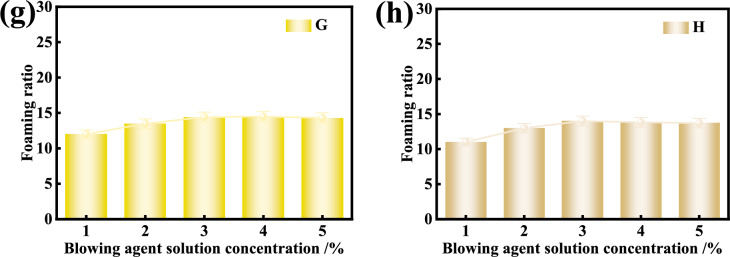
Variation of foaming ratio with blowing agent solution concentration.

Fig. 9 shows the foaming ratios of eight kinds of foaming agents tested at different concentrations of 1–5%. The foaming ratios of all foaming agent solution increases gradually with the increase of the solution concentration, reaching the maximum almost at the concentration of 3% and then tend to be stable. ANOVA results (p > 0.05) confirm no significant difference when the blowing agent solution concentration is ranging between 3%, 4%, and 5%. Generally, all foaming agents but the foaming agent B have the maximum foaming ratio greater than 12, which is consistent with the highest stabilised surface tension of foaming agent B among the tested foaming agents.

It should be noted that the foam generated during EPB shield tunnelling is under face pressure, while the laboratory test is generally carried out under standard atmospheric pressure. Since the FER varies with the imposed pressure, the amount of foaming agent stock liquid required under the excavation chamber is different from that in the lab. The FER in the excavation chamber can be derived from that under atmospheric conditions as below.

3 $${V}_{A,P}={V}_{A,0}\times \frac{{p}_{atm}}{p}$$

4 $${FER}_{0}=\frac{{V}_{F,0}}{{V}_{L}}=\frac{{V}_{L}+{V}_{A,0}}{{V}_{L}}=1+\frac{{V}_{A,0}}{{V}_{L}}$$

5 $${FER}_{p}=1+\frac{{V}_{A,p}}{{V}_{L}}=1+\frac{{V}_{A,0}\frac{{p}_{atm}}{p}}{{V}_{L}}=1+\left({FER}_{0}-1\right)\frac{{p}_{atm}}{p}$$

Where: ${V}_{A,P}$ is foam volume under pressure *p*; ${V}_{A,0}$ is the volume of foam under atmospheric pressure; ${V}_{L}$ is the volume of foaming agent solution; ${\mathrm{p}}$ is the excavation chamber pressure of the shield machine; ${p}_{atm}$ is standard atmospheric pressure; ${FER}_{0}$ is the foam expansion ratio measured at standard atmospheric pressure; ${FER}_{p}$ is the foam expansion ratio under the excavation chamber pressure.

According to the experimental results, it can be inferred that: The optimal foaming concentration of the foaming agent solution is 3%. The foaming ratio of the foaming agent reaches the optimal foaming concentration is uneven, so it is necessary to give a reasonable foaming ratio range. When the *FER* is 10–20, the foaming ratio of the foam can meet the construction needs^38^. If the FER value is too high (such as more than 20), it may lead to too many bubbles and unstable foam structure. When the FER value is too low, it may not be able to achieve the desired conditioning effect. Therefore, the foam of blowing agent is the most suitable in this range, which can take account of the stability and economy aspects of the foam. The selection of this range ensures that the foam has sufficient foaming formation capacity, while avoiding waste and unnecessary costs, and improving operability and efficiency during construction. Combined with considerations of the economy and the conversion relationship as defined in Eq. (5), it is suggested that when the optimal foaming concentration is reached, the reasonable foaming ratio of a foaming agent under standard atmospheric pressure in metro shield construction shall be greater than 12.

### Comparison of foaming capability of various foaming agents

**Fig. 10:**
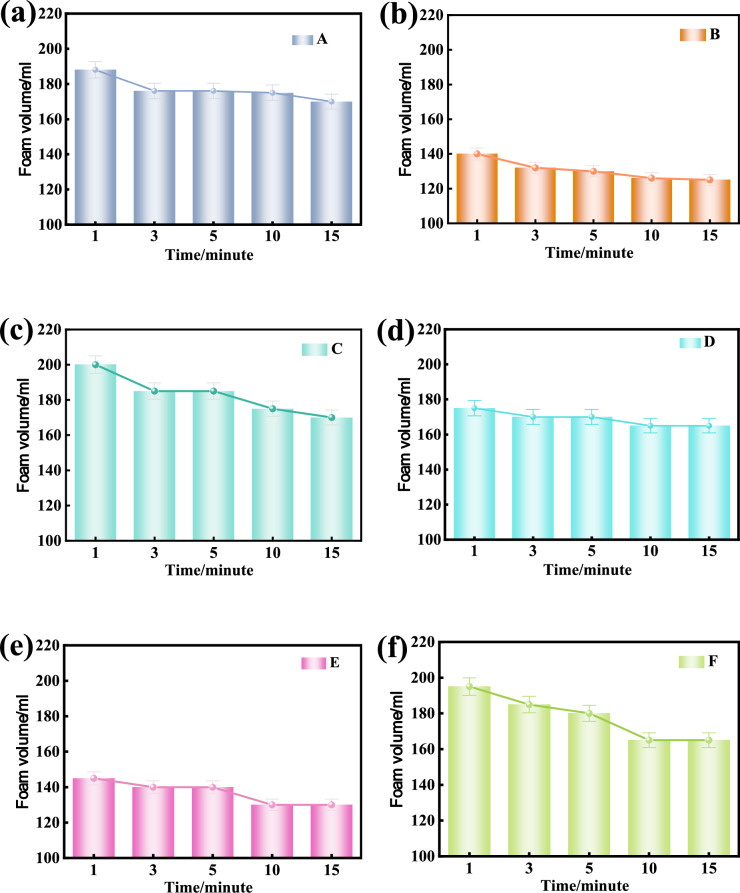

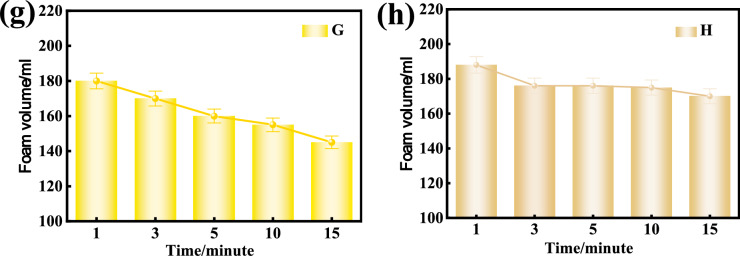
Decay of foam volume with time for various foam solutions with 3% concentration.

Fig. 10 shows the decay of foam volume for various foam solution at the optimal concentration of 3% from the tests with the Roche foam meter, reflecting the foaming capability of foaming agent and foam stability. In each test, 200 ml solution was dropped into the Roche foam meter for testing. It can be seen that the volume of foam from the foaming agent A-H decreases with time. The foaming capability of foaming agent B and E is low, which is corresponding to the high surface tensions of foaming agent B and E after reaching the critical micelle concentration. The foam from the foaming agent G bursts most obviously within 15 min, while the foaming agent A is superior in terms of both foaming capability and foam stability. By comparing the foaming volume and foam decay rate for the 8 kinds of foaming agents, the foam volume from the Roche foam meter greater than 150 ml within 15 min may be acceptable.

### Foam stability based on half-life test

**Fig. 11:**
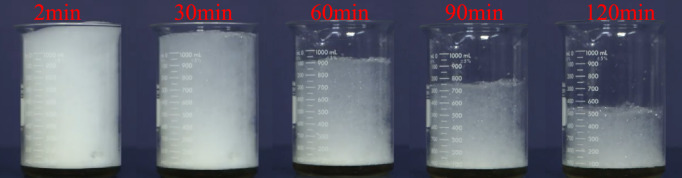
Foam burst with time.

Fig. 11 shows the test results of foam burst progressively at times of 2 min, 30 min, 60 min, 90 min and 120 min after generation. Within the first 2 min, the foam was dense in a stable state and foam burst was tiny. When foam was just produced, gas was dispersed in the liquid medium, forming bubbles surrounded by liquid films. At first, because the bubbles were very small and the liquid films were thick, the foam burst appeared tiny. Due to the existence of surface tension, the foam remained structurally stable and relatively resistant to rupture at this stage. After 30 min, the foam at the top became large and thinner, and the foam volume gradually decreased. This phenomenon occurred when many small bubbles merged into larger ones, which is a process known as Ostwald ripening or foam coarsening. In this process, the liquid in the small bubble moves to the large bubble, the total surface area of the foam is continuously reduced, the liquid film is thinner, the foam density is reduced, and the foam volume is reduced. Leaving the foam up to 120 min, the action of gravity caused the liquid in the foam to drain out. This phenomenon caused the foam structure to weaken, and the bubbles gradually lose their support. In some cases, evaporation of the liquid phase can further cause the foam to thin and burst. The foam volume dropped to almost half and the foam was very thin. Overall, the observed phenomenon is due to a combination of coalescence, drainage, evaporation, and bubble rupture, resulting in a reduction in foam volume and thinning over time.

**Fig. 12:**
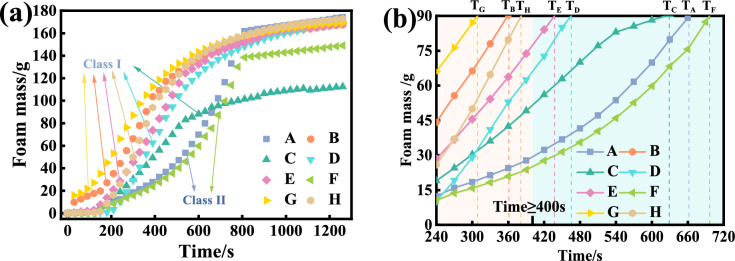
Comparison of foam fading quality with time and half-life.

Fig. 12(a) shows the variation of foam fading quality of the 8 foaming agent solution at the concentration of 3%. Taking 180 g foam for testing, when the fading foam mass reached 90 g, the foam survival time was the foam half-life. The decay rate of the eight foams was fast (accelerating decay) at the beginning and then became slow (gradual decay). Fig. 12(b) shows the foam accelerating decay to the half-life quality. From the Figure (a), the foam accelerating decay can be classified into two types, i.e. linear decay (Class Ⅰ) and concave decay (Class Ⅱ). Generally, the foam with concave decay has longer half-life. The longer the half-life, the better the foaming agent is. Therefore, the foaming agent A and F are better than other foaming agents, especially the foaming agent B and G with much shorter half-life, which is generally consistent with test results from the Roche foam meter. In combination with the recommended selection criteria from the surface tension, foaming ratio and that from the Roche foam meter, the half-life of the foaming agent used for soil conditioning shall be greater than 400 s. Based on this criterion, the foaming agent A, F, C and D could be satisfactory.

### Selection criteria of a foaming agent

The selection criteria and optimum values for the foaming agent were established to form a comprehensive and multi-stage evaluation framework, ensuring the selected agent excels in key aspects of foam performance. This framework is designed to assess: (1) the fundamental efficiency of foam formation, governed by the agent’s ability to lower surface tension at the Critical Micelle Concentration (CMC); (2) the volumetric expansion capability, which determines the yield and “dryness” of the foam; (3) the short-term stability against rapid drainage and coalescence after generation, as measured by a standardized method; and (4) the long-term structural persistence, reflected in the foam’s half-life and its decay pattern. The specific thresholds and optimum values summarized below are consequently derived from a synthesis of surfactant theory, recognized industry standards^39^, and iterative experimental validation tailored to our specific application. Based on the above comparative analyses, the selection criteria of foaming agent and its solution are summarised below:

1. The surface tension should be less than 40 mN/m at the critical micelle concentration and the optimum solution concentration is 3%;
2. The foaming ratio of foaming agent solution should be greater than 12 under atmospheric pressure;
3. The foam volume from the Roche foam meter should be greater than 150 ml within 15 min after foam generation; and
4. The half-life of the foam shall be greater than 400 s and the concave decay type of foam is preferred.

## Mechanical performance evaluation of foam conditioned sandy soil and validation of foam selection criteria

### Target performance of conditioned sandy soils

(i) obtain the appropriate slurry fluidity to ensure that the excavated sand can enter the excavation chamber smoothly through the cutter head openings, with a general cutter head opening ratio of 30–40%^19^. In this study, the slurry fluidity was quantitatively evaluated using the slump test. This method, while not governed by a single universal standard, is a widely recognized empirical tool in geotechnical and construction engineering for assessing the workability of soil-binder mixtures and debris-flow slurries. The slump value exhibits a strong inverse correlation with key rheological parameters such as yield stress and plastic viscosity, making it a practical and effective indicator for ensuring the conditioned soil achieves a pumpable, slurry-like consistency suitable for EPB operation. At the same time, the excavated muck is smoothly discharged by the screw conveyor. Therefore, the conditioners need to be injected from the cutter head and bulkhead, and if necessary, at the screw conveyor^12^, (ii) achieve homogeneous plastic material for transfer of the bulkhead pressure to the tunnel face uniformly. Thus, it is necessary to continuously stir the excavated muck with the injected conditioners^40,41^; (iii) reduce the permeability of the excavated muck to prevent gushing along the screw conveyor^42,43^, and (iv) reduce the friction angle of the excavated muck, thereby reducing the cutter torque, cutter and screw energy consumption, and the wear of cutters and the cutterhead^44,45^.

Based on comprehensive tests^46^ recommended the following mechanical target performance parameters of a conditioned soil as shown in Table 1: The flow plasticity and permeability of the conditioned soil were evaluated mainly by means of slump (ideal value is 150–200 mm), undrained vane shear strength (3–7 kPa), permeability coefficient (k < 10^–5^ m/s).

**Table 1:** Recommended soil index properties for EPB-shield without soil conditioning.

Muck parameter	Parameter ranges
Osmotic coefficient	K < 10^–5^ m/s
Muck fluidity	liquidity index IL = 0.4–0.75
Screw conveyor excavation muck	liquidity index IL = 0.6–0.75
Friction angle of the excavated muck	15° < φ < 25°

**Fig. 13:**
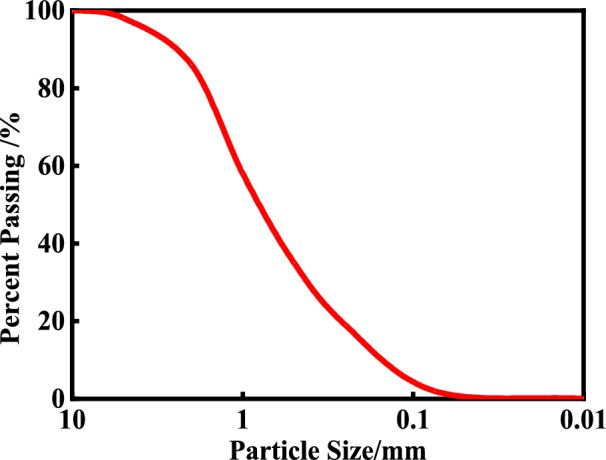
Grain size distribution curve of poorly graded sand (SP).

Three foaming agents (A, B and C) were initially selected from the eight kinds of foaming agent mentioned above to assess the soil conditioning effect and validate the selection criteria of foaming agent. Foaming agent A and C were chosen for testing given their characteristics of the concave decay type and the linear accelerating decay type, respectively. Foaming agent B was not recommended after further assessment based on the above selection criteria. Sand samples were collected from typical water-rich sand strata from metro lines in Nanchang City, China. The typical gradation curve of the sand is shown in Fig. 13, With D10 being 0.15 mm; the coefficient of uniformity (Cu) = 2.1 and the coefficient of curvature (Cc) = 0.9. These values confirm that the sand is poorly graded (SP) according to ASTM D2487. Gravel layer of the water-rich sand strata in the metro shield tunnel horizon was also sampled, and the excavation muck was improved and compared after conditioning with the selected foaming agents at the optimal foaming concentration.

### Slump comparison of conditioned sandy soil for typical foaming agent

For EPB shield tunnelling, Foam Injection Ratio (FIR) is usually used to characterize the foam dosage, namely:

6 $$\mathrm{FIR} = \frac{{\mathrm{V}}_{\mathrm{f}}}{{\mathrm{V}}_{\mathrm{s}}}$$

where ${V}_{f}$ is the foam injection volume; ${V}_{s}$ is the volume of excavated soil by the cutterhead. Usually, a bulking factor of 1.2–1.4 is used for calculating the volume of excavated soil and 1.2 is preferable for sandy soil^47,48^.

**Fig. 14:**
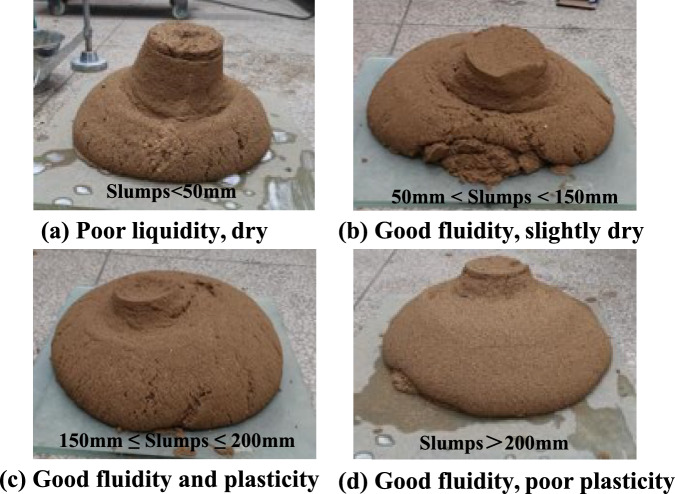
Conditioned soil morphology under different slumps.

**Fig. 15:**
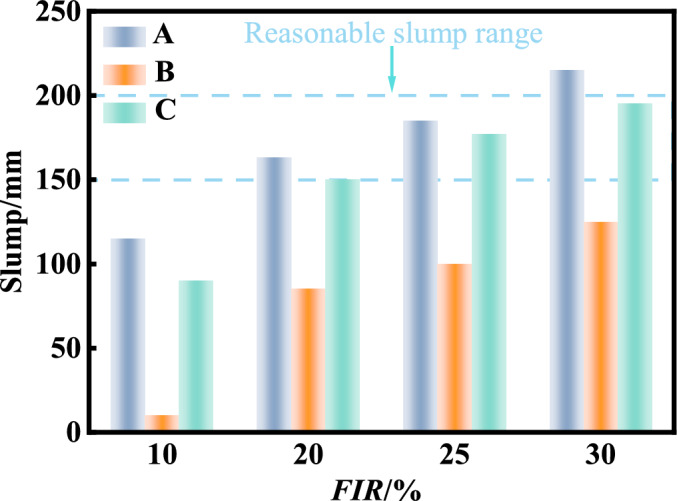
Slump of excavation muck under different foam injection ratio.

In this study, only foam was used for soil conditioning. In each test, a estimated amount of excavated muck was dried, and water was then added to achieve the moisture content of 5%^49^. In this experimental phase, a relatively low initial moisture content of 5% was adopted for the remoulded soil samples. This condition was intentionally selected to establish a benchmark conditioned state in a dried soil, allowing for a comparison of the efficacy of the different foaming agents by minimizing the confounding influence of variable initial water content. It should be noted that this condition does not replicate the in-situ state of water-rich strata, where a higher initial moisture content would be expected to significantly alter the soil’s consistency and foam demand^39^. Then, 10%, 20%, 25%, and 30% of foams were added to the remoulded soil. The typical morphology of the conditioned soil with varying slumps is shown in Fig. 14. The slump of the conditioned soil with varying foam injection ratio is shown in Fig. 15. It can be seen that the volumetric injection ratio of 20–25% for foam A and 25–30% for foam C can achieve the ideal slump of 150–200 mm, while the foam B could not achieve the ideal slump even with 30% volumetric injection ratio. This has confirmed that the foaming agent B is not suitable for sandy soils and foam (A) of the concave decay type is preferred to foam (C) of the linear accelerating decay type .

### Permeability comparison of conditioned sandy soil

**Fig. 16:**
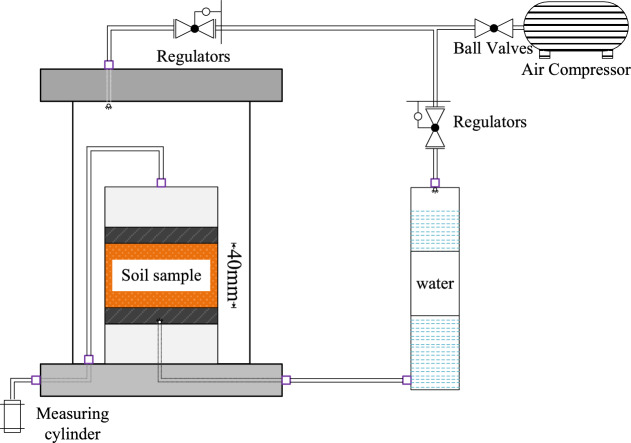
Experimental equipment of pressurizing permeability.

**Fig. 17:**
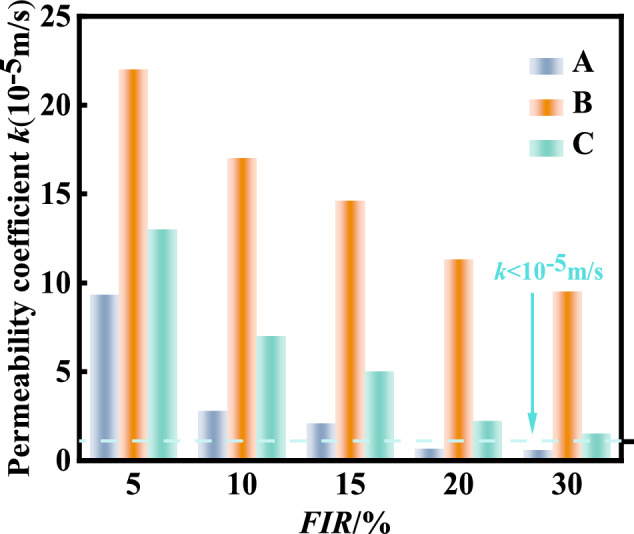
Variation of soil permeability coefficient with FIR for different foaming agent.

To assess the permeability of conditioned sand for the foaming agents A, B and C under EPB excavation chamber pressure, the permeability test set-up is shown in Fig. 16. Foam A, B and C were generated by using the foaming agents A, B and C, respectively, with the optimal foaming concentration of 3%. During each test, the confining pressure of the soil sample was controlled to be 200 kPa and the osmotic water pressure to be 150 kPa. The resulting permeability coefficient of conditioned soil changed with the varying foam injection ratio is shown in Fig. 17. It can be seen that: (1) the permeability coefficient of the excavation muck decreases remarkably with increase of FIR, especially for foam A and C; (2) The volumetric injection ratio greater than 20% for foam A can achieve the permeability coefficient *k* < 10^–5^ m/s, while the foam C and B could not achieve the permeability coefficient even with 30% injection ratio. This has confirmed that the foaming agent A is suitable for sandy soil. For the foaming agent C and B, probably bentonite slurry should be further added to reduce the permeability coefficient of sandy soils.

### Comparison of undrained shear strength of conditioned sandy soil

**Fig. 18:**
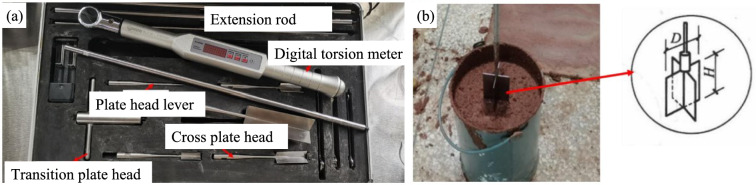
Undrained shear strength test for conditioned soil with a mini shear vane:(**a**) cross plate shear instrument (**b**) test process diagram.

**Fig. 19:**
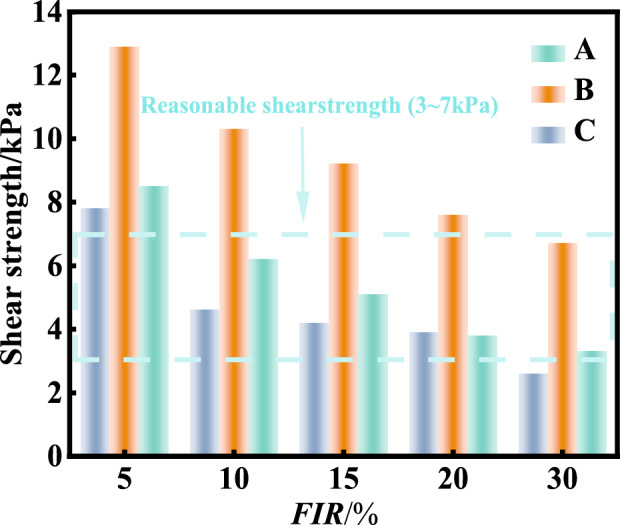
Variation of undrained shear strength with foam injection rate for different foams.

To evaluate the undrained shear strength of conditioned soil, a mini shear vane was used. The D and H follow the requirement of being at least 10 times the diameter of the largest particle size of sands, as shown in Fig. 18. The desired undrained shear strength is 3–7 kPa^50^. Fig. 19 illustrates the change of the undrained shear strength of conditioned sand for the foaming agent A, B and C with varying injection ratios. It can be seen that (1) the undrained shear strength decreases with the increase of FIR for each foaming agent; (2) The volumetric injection ratios of 10–30% for foam A and foam C yield the undrained shear strength of 3–7 kPa. However, foam B could not achieve the undrained shear strength of less than 7 kPa until the volumetric injection ratio reaching 30%. This has confirmed that the foaming agent A and C are satisfactory, while the foaming agent B is not .

## Conclusions

In this study, a series of tests have been carried out on the properties of eight kinds of foaming agent solution and foam, including the surface tension, foaming ratio, foaming capability and foam half-life etc. Furthermore, three representative foaming agents were selected for investigation of the slump, permeability and undrained shear strength of the conditioned water-rich sandy soil. The main findings of these investigations are summarised as follows:

1. the selection criteria of foaming agent and its solution are: a) The surface tension should be less than 40 mN/m at the critical micelle concentration; The optimum solution concentration is 3%; c) The foaming ratio of foaming agent solution should be greater than 12 under atmospheric pressure; d) The foam volume from the Roche foam meter should be greater than 150 ml within 15 min after foam generation; e) The half-life of the foam shall be greater than 400 s; and f) The concave decay type of foam is preferred.
2. The surface tension of foaming agent solution can be accurately measured by the OCA measuring instrument. All foaming agent solution has a critical micelle concentration beyond which the surface tension will drop gradually, and the surface tension tends to be stabilised at the concentration of 2–3%;
3. The foaming capability and stability can be measured by the Rothschild foam apparatus. The foam volume shall not be less than 150 ml within 15 min after foaming with the Rothschild foam apparatus.
4. The foam half-life can be measured with Roche foam meter. The foam half-life is required to be greater than 400 s.
5. The conditioning tests for typical water-rich sand in Nanchang with the representative foaming agent and solution have confirmed that the above criteria and test methods for foaming agent selection are appropriate. It has found that the desired slump of 150–200 mm, permeability coefficient k < 10^–5^ m/s, and undrained shear strength of 3–7 kPa for the conditioned soil are workable indices for EPB shield TBM tunnelling through sands in Nangchang.

## Data Availability

Some or all data, models, or code that support the findings of this study are available from the corresponding author upon reasonable request.
